# The fermentation optimization for alkaline protease production by *Bacillus subtilis* BS-QR-052

**DOI:** 10.3389/fmicb.2023.1301065

**Published:** 2023-12-19

**Authors:** Biao Sun, Kai Zou, Yingqing Zhao, Yinfang Tang, Fuming Zhang, Weijing Chen, Xiaoting Tang, Chenran Chang, Yan Zheng

**Affiliations:** ^1^Zhejiang Cheery Bio-Technology Co., Ltd., Jiaxing, China; ^2^College of Advanced Materials Engineering, Jiaxing Nanhu University, Jiaxing, China; ^3^Zhejiang Institute of Advanced Technology Chinese Academy of Sciences, Jiaxing, China; ^4^Sinochem Linyi Crop Nutrition Co., Ltd., Linyi, China

**Keywords:** *Bacillus subtilis*, alkaline protease, fermentation optimization, single-variable experiments, response surface methodology

## Abstract

**Introduction:**

Proteases exhibit a wide range of applications, and among them, alkaline proteases have become a prominent area of research due to their stability in highly alkaline environments. To optimize the production yield and activity of alkaline proteases, researchers are continuously exploring different fermentation conditions and culture medium components.

**Methods:**

In this paper, the fermentation conditions of the alkaline protease (EC 3.4.21.14) production by *Bacillus subtilis* BS-QR-052 were optimized, and the effect of different nutrition and fermentation conditions was investigated. Based on the single-variable experiments, the Plackett–Burman design was used to explore the significant factors, and then the optimized fermentation conditions, as well as the interaction between these factors, were evaluated by response surface methodology through the Box–Behnken design.

**Results and discussion:**

The results showed that 1.03% corn syrup powder, 0.05% MgSO_4_, 8.02% inoculation volume, 1:1.22 vvm airflow rate, as well as 0.5% corn starch, 0.05% MnSO_4_, 180 rpm agitation speed, 36°C fermentation temperature, 8.0 initial pH and 96 h incubation time were predicted to be the optimal fermentation conditions. The alkaline protease enzyme activity was estimated to be approximately 1787.91 U/mL, whereas subsequent experimental validation confirmed it reached 1780.03 U/mL, while that of 500 L scale-up fermentation reached 1798.33 U/mL. This study optimized the fermentation conditions for alkaline protease production by *B. subtilis* through systematic experimental design and data analysis, and the activity of the alkaline protease increased to 300.72% of its original level. The established model for predicting alkaline protease activity was validated, achieving significantly higher levels of enzymatic activity. The findings provide valuable references for further enhancing the yield and activity of alkaline protease, thereby holding substantial practical significance and economic benefits for industrial applications.

## 1 Introduction

Proteases can hydrolyze proteins into peptides, short-chain peptides and amino acids, and are categorized into neutral proteases, acid proteases and alkaline proteases according to their optimum reaction pH values. Among these, alkaline protease is the most highly demanded enzyme preparation in the market, with applications spanning various production sectors, including food processing, pharmaceuticals, the detergent industry, textiles, leather processing, and feed additives (Ito et al., 1998). So far, There are dozens of commercially produced alkaline proteases available, with the main source of production being the alkaline proteases (EC 3.4.21.14) produced through the metabolic activity of *Bacillus subtilis* (Sharma et al., 2017).

*B. subtilis*, one kind of Gram-positive bacteria (Vanitha et al., 2014), distributes widely on earth, like air and water, as well as guts of human beings and animals. When its survival environment, such as the acidity, temperature, humidity and nutrient substance, etc., has dramatically changed, it will generate spore to tackle these changes. During the stable phase of fermentation, a significant amount of proteases is synthesized as a result of substrate induction (Singh et al., 2015). *B. subtilis* has been scientifically validated to meet the requirements for biological safety (Harwood et al., 2018), which allows it to be legally approved by the Chinese Ministry of Agriculture for use as a feed additive. As a member of the probiotic bacteria, *B. subtilis* is utilized for the production of a microbial ecological formulation (Morikawa, 2006; Hashem et al., 2019). This eco-friendly, naturally derived, and non-polluting formulation has exhibited significant effectiveness, leading to its extensive application across various industries, including manufacturing, agriculture, pharmaceuticals, healthcare, and aquaculture.

Although *B. subtilis* exhibits good enzymatic activity, the existing strains are unable to meet the increasing demands driven by societal development. Consequently, researchers commonly employ methods such as wild strain isolation and screening, mutagenesis breeding and genetic engineering to enhance alkaline protease activity, stability, and yield. Through targeted modification and screening of strains, they aim to obtain optimal strains. The evaluation of these strains typically relies on the alkaline protease content and enzyme activity achieved through microbial fermentation. For example, Rekik achieved an alkaline protease enzyme activity of 9,000 U/mL in *B. subtilis* through the optimization of the culture medium (Rekik et al., 2019). Similarly, other researchers have also optimized the fermentation conditions for different alkaline protease-producing strains, resulting in significant improvements in alkaline protease activity (Qureshi et al., 2011; Pant et al., 2015).

Optimization of variables is a crucial aspect of scientific exploration. Typically, various approaches are employed, including single-variable experiments, orthogonal experimental design (Box and Wilson, 1992), response surface methodology (RSM) (Khuri and Mukhopadhyay, 2010), and so on. Among these, RSM is a widely used method in experimental design and statistical modeling. It is primarily employed to study the influence of multiple factors on a specific response variable and to identify operating conditions that optimize the response variable. Characteristics of RSM include a comprehensive examination of the impacts of multiple factors, the description of the relationship between factors and the response variable through mathematical models, and the determination of optimal operating conditions through the design of a series of experiments. Furthermore, RSM emphasizes the interactions between factors and provides visualization tools, such as response surface plots, to intuitively display these relationships. Through statistical inference, the significance and reliability of the model can be assessed, thereby providing support for the ultimate optimization decisions.

Research has shown that alkaline proteases possess strong hydrolytic and alkali-resistant capabilities, as well as significant thermal stability (Mei and Jiang, 2005). Currently, most of these alkaline proteases used in the industry rely on imports, which greatly increases the production costs of enterprises. This study focuses on the alkaline protease production ability of *B. subtilis* BS-QR-052, which has been purified from the environment. The main objective of the research is to optimize the composition of the fermentation medium based on conventional and cost-effective production fermentation media. This involves fine-tuning the culture conditions to enhance the alkaline protease yield and enzyme activity in the RSM. Ultimately, the study aims to establish a solid theoretical foundation for the future industrial applications of this enzyme and provide solutions to the problem of high enzyme production costs in the industry.

## 2 Materials and methods

### 2.1 Microbial strain and culture medium

*B. subtilis* BS-QR-052, isolated from environmental samples in the Key Microbiological Laboratory of Zhejiang Cheery Co., Ltd., was identified as a member of the *Bacillus genus* through phylogenetic analysis based on the 16S rDNA (Woese and Fox, 1977) sequence and the CVTree3 algorithm (Qi et al., 2004). Before use, it was stored in a glycerol tube at −40°C following lyophilization. Three main types of culture media, namely solid slant medium (in g/100 mL): 1.0 peptone, 0.5 yeast extract, 1.5 NaCl, and 1.5 agar at pH 10.0, liquid seed medium (in g/100 mL): 1.0 beef paste, 1.5 glucose, and 0.5 NaCl at natural pH, and basal fermentation medium (in g/100 mL): 0.5 glucose, 1.0 peptone, 0.05 NaCl at pH 10.0, were utilized throughout the entire research process. As designed, each research step included three biological replicates. All chemical reagents and culture medium nutrients were purchased from Shanghai Aladdin Biochemical Technology Co., Ltd.

### 2.2 Cultivation conditions

Fifty milliliters of liquid seed medium was incubated overnight at 37°C and 200 rpm to obtain a primary seed culture in an Erlenmeyer flask in the incubator shaker (HZQ-F160A, Shanghai Bluepard Instruments Co., Ltd.). For the secondary seed culture, 5% of the primary seed culture was inoculated into a new Erlenmeyer flask containing a liquid seed medium, following the same cultivation method as the primary seed culture.

### 2.3 The viable count and enzyme activity assay of basal fermentation

A basal fermentation medium was used to prepare a 50 L fermentation broth (BLBIO-50-500-2000, Shanghai Bailun Biological Technology Co., Ltd., China), with the initial pH adjusted to 7.0. The inoculation was performed using a flame, with 5% of the secondary seed culture inoculated into the fermentation broth. The fermentation was carried out at a temperature of 40°C, an airflow rate of 1:1.2 vvm (volume of air per volume of medium), and an agitation speed of 150 rpm for 120 h. Samples were taken every 2 h to monitor the viable cell count and alkaline protease production in the fermentation broth. After reaching the stable phase of fermentation, a certain amount of fermentation broth was taken and centrifuged at 7155 × g for 10 min. The supernatant was collected to measure the alkaline protease activity.

The National Standard of PRC (GBT 23527-2009) was employed to measure the enzyme activity of alkaline proteases (Huang et al., 2020). Here, One unit of enzyme activity is defined as the amount of enzyme that liberates 1 μg tyrosine from casein as a substrate at 45°C at pH 10.0 per minute. The 1% (10 g/L) casein solution was used as the reaction substrate. A standard curve was constructed by preparing L-tyrosine standard solutions with concentrations of 0, 10, 20, 30, 40, 50, 60, 70, and 80 μg/mL. A blank solution without L-tyrosine was prepared in a separate tube. According to the proposal, Folin’s reagent was used to react with L-tyrosine under alkaline conditions to produce a blue compound, and the absorbance of this product was measured at a wavelength of 680 nm. The enzyme activity of all samples is calculated by the following formula:


$$X=\frac{A\times V\times 4\times n}{m}\times \frac{1}{10}$$

in this equation: *X* = The enzyme activity of samples, (u/g). *A* = Enzyme activity of the final dilution compared to the standard curve, (u/mL). *V* = The volume of the volumetric flask used to dissolve the samples, (mL). 4 = The total volume of the reaction mixture is 4 mL. *n* = The dilution factor of enzyme liquid of alkaline proteases. *m* = The mass of the samples, (g). 10 = The reaction time is 10 min.

### 2.4 One variable at a time experiments: physicochemical determinants for alkaline protease production

Based on the single-variable experiment, the effect of carbon sources, nitrogen sources, and metal ions on the production of alkaline protease by *B. subtilis* was studied in a basal fermentation medium. All carbon and nitrogen sources were tested at 1% and 0.5%, respectively. The carbon sources included sucrose, lactose, maltose, glucose, corn starch, maltodextrin, and cornmeal. The nitrogen sources included soybean meal, peptone, urea, yeast extract, beef paste, soybean cake powder, corn steep powder, NH_4_Cl, and bran. Metal ions such as 0.05% CaCl_2_, MgSO_4_, MnSO_4_, CuSO_4_, FeCl_3_, and K_2_HPO_4_ were also added by turns. The influence of these three components on alkaline protease production was investigated while keeping other culture conditions constant. The fermentation process lasted for 96 h at a temperature of 38°C, with an inoculation volume of 5%, pH set at 7.0, agitation speed of 150 rpm, and an airflow rate of 1:1 vvm. Then, the supernatant was collected by centrifugation, and the enzyme activity was measured to determine the most suitable combination for alkaline protease production by *B. subtilis*.

A 10 L glass fermenter (JD-GAFM-10L, Beijing Jiade Precision Technology Co., Ltd., China) with 70% liquid volume was used for fermentation experiments employing a basal fermentation medium. The fermentation was conducted under the following conditions: a temperature of 38°C, an inoculation volume of 5%, an initial pH of 7.0, an agitation speed of 150 rpm, and an airflow rate of 1:1 vvm. Single-variable experiments were performed to investigate the effects of fermentation temperature (28, 32, 36, 40, 44, 48%), agitation speed (140, 150, 160, 170, 180, 190, 200, 220 rpm), inoculation volume (2, 4, 6, 8, 10, 12, 14%), initial pH (6.0, 6.5, 7.0, 7.5, 8.0), and airflow rate (1:0.6, 1:0.8, 1:1.0, 1:1.2, 1:1.4, 1:1.6 vvm) on the fermentation process. After 96 h of fermentation, the supernatant was collected by centrifugation, and the enzyme activity was measured.

### 2.5 Multiple variables at a time experiments: optimal combination of physicochemical factors for alkaline protease production

Plackett and Burman (1946) designs were employed for the rapid screening of significant factors influencing the experimental process, with each variable coded at two levels, −1 and +1. Then, the factors that remarkably influence fermentation were screened out for Box–Behnken design (Ferreira et al., 2007), with each factor tested in three gradients, −1, 0 and +1, to conduct the RSM (Khuri and Mukhopadhyay, 2010).

### 2.6 The scale-up test of alkaline protease fermentation

Using an optimized fermentation medium, a 500 L fermentation broth (BLBIO-50-500-2000, Shanghai Bailun Biological Technology Co., Ltd., China) was prepared. The fermentation process was carried out under optimized conditions for a cultivation period of 96 h. Samples were collected every 2 h to measure the viable cell count and alkaline protease enzyme activity in the fermentation broth.

### 2.7 The software used for statistical analysis

Microsoft 365 (Microsoft Corporation, United States) is employed for the compilation of all experimental data and tables. Origin Pro (OriginLab Corporation, United States) is utilized for generating trend charts and bar graphs. Design Expert 11(Stat-Ease Corporation, United States) is applied for Plackett–Burman design, Box–Behnken design, and RSM. All exported graphs are assembled and refined in Adobe Illustrator (version 26.0, Adobe Corporation, United States).

## 3 Results

### 3.1 The viable count and alkaline protease activity in the basal fermentation medium

**Figure 1:**
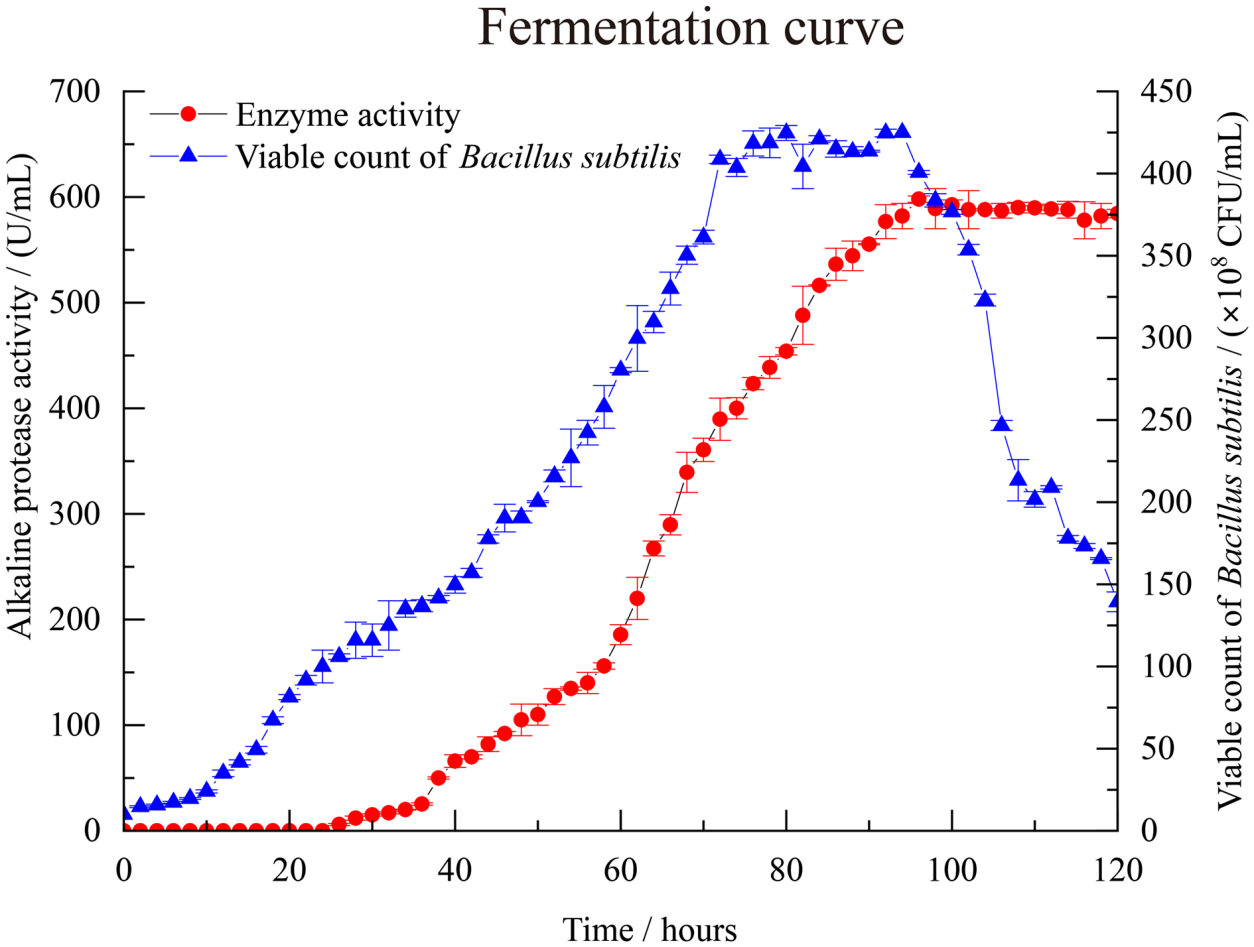
*Bacillus subtilis* BS-QR-052 growth and alkaline protease production in 50 L laboratory scale fermenter. Values are the mean of three replicas ǂ standard error or standard deviation.

In Figure 1, the viable cell count of *B. subtilis* increased slowly within the first 10 h, and no detectable alkaline protease activity was observed according to the standard curve of the L-tyrosine (*y* = 0.01*x* + 0.0035, *R*^2^ = 0.9998) in Supplementary Figure S1. At 26 h of cultivation, alkaline protease activity of 6 U/mL was detected, accompanied by an increase in the cell count of *B. subtilis*, indicating rapid proliferation of the bacterial cells entering the logarithmic growth phase. In the early stages of fermentation, the enzyme production rate was relatively slow. When the fermentation time reached 96 h, the alkaline protease activity reached its highest value of 598 U/mL. Subsequently, there was no significant change in enzyme production with increasing fermentation time. This may be due to the fact that in the late stages of fermentation, substrate depletion becomes severe, leading to a decline in cell viability and enzyme production capacity. Therefore, the fermentation time for *B. subtilis* to produce alkaline protease is determined as 96 h.

### 3.2 The optimization of nutritional composition and fermentation conditions

**Figure 2:**
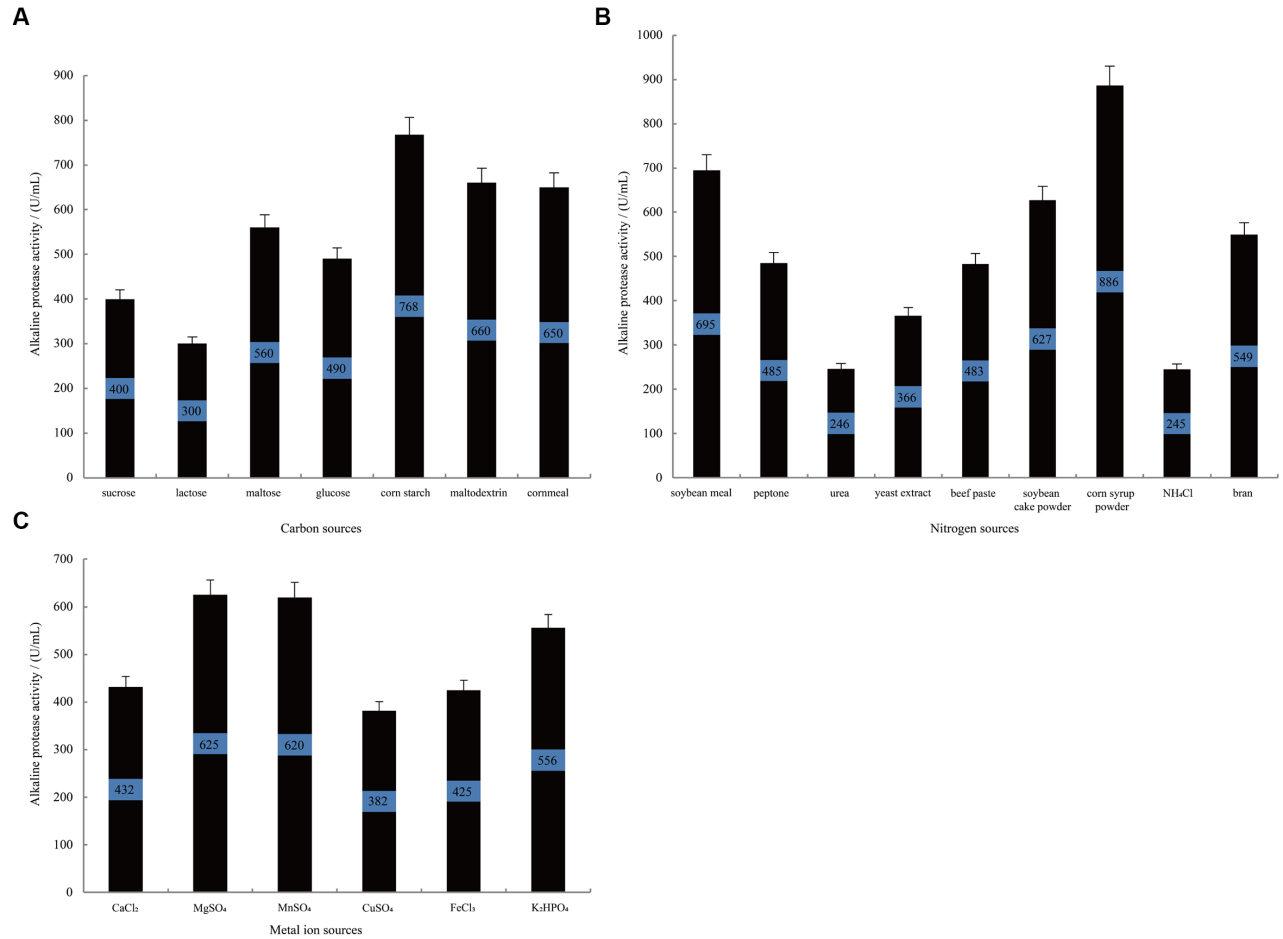
The single-variable experiments conducted to investigate the nutritional components. The effect of carbon source **(A)**, nitrogen source **(B)**, and metal ion **(C)** on the alkaline protease production by *B. subtilis* BS-QR-052. Values are the mean of three replicas ǂ standard error or standard deviation.

Figure 2A illustrates that when using corn starch as the carbon source, *B. subtilis* exhibits the highest alkaline protease activity during the stationary phase, reaching 768 U/mL. This activity is significantly superior to that observed when using cornmeal (*p*-vlaue = 0.02) and maltodextrin (*p*-vlaue = 0.03) as alternative carbon sources. Similarly, when investigating different nitrogen sources (Figure 2B), the group supplemented with corn syrup powder demonstrated the highest alkaline protease activity, reaching 886 U/mL, which was significantly higher than the enzyme activity when soybean meal was used as a nitrogen source (*p*-value = 0.02). Following closely are soybean cake powder and bran, while urea and NH_4_Cl show the lowest activity. Furthermore, the influence of metal ions on alkaline protease production was studied. As depicted in Figure 2C, the groups treated with MgSO_4_ and MnSO_4_ display the highest alkaline protease production, reaching 625 U/mL and 620 U/mL, respectively. In contrast, the groups treated with K_2_HPO_4_ and CaCl_2_ show relatively lower protease production, with *p*-values of 0.058 and 0.002 against that of MgSO_4_, respectively. These results collectively indicate that corn starch and corn syrup powder are more suitable as carbon and nitrogen sources to enhance the alkaline protease production by *B. subtilis*. Additionally, the presence of Mg^2+^ and Mn^2+^ positively impacts alkaline protease proliferation and metabolism in *B. subtilis*.

**Figure 3:**
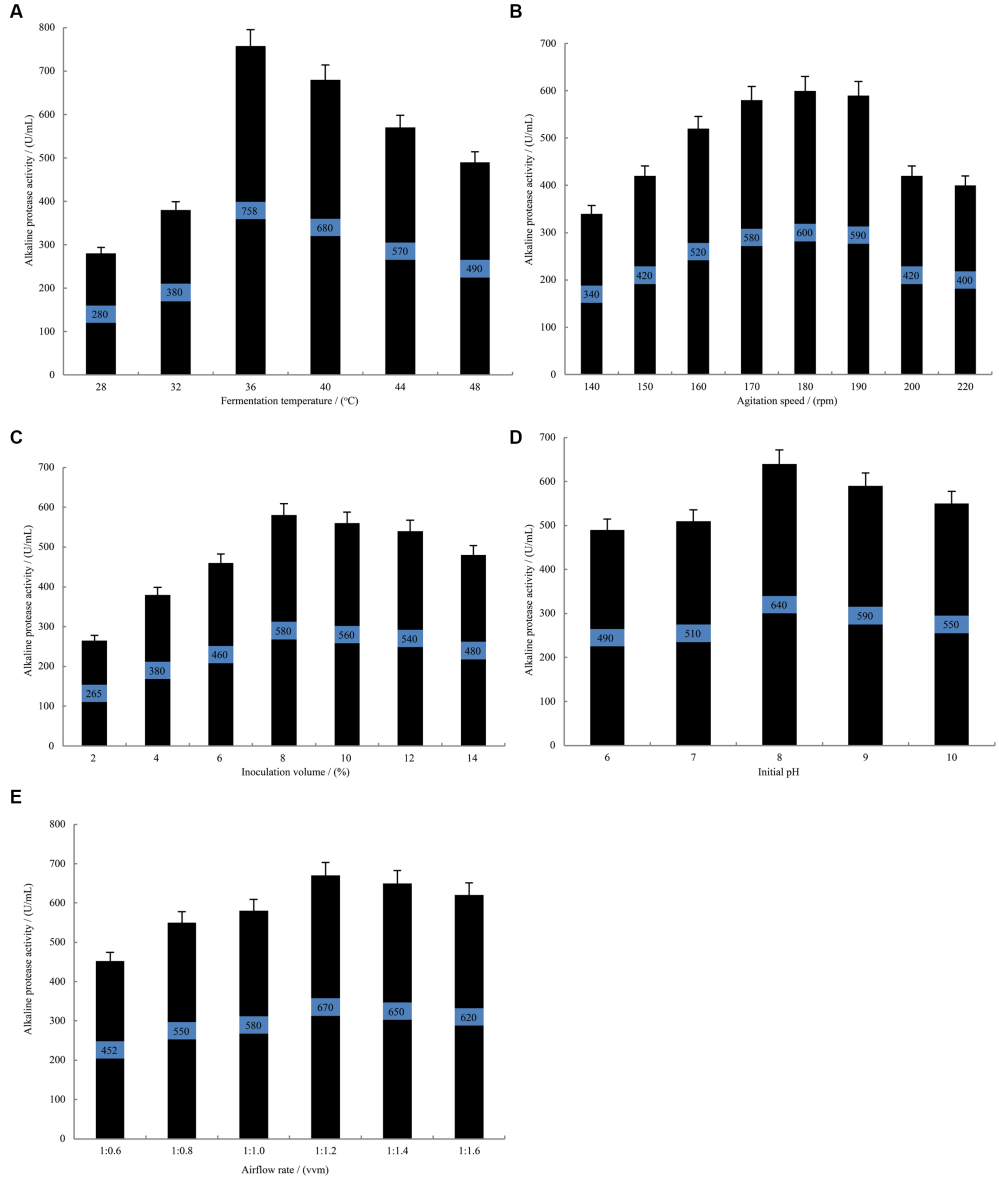
The single-variable experiments conducted to evaluate the fermentation conditions. The effect of fermentation temperature **(A)** agitation speed **(B)** inoculation volume **(C)** initial pH **(D)** airflow rate **(E)** on the alkaline protease production by *B. subtilis* BS-QR-052. Values are the mean of three replicas ǂ standard error or standard deviation.

Single-variable experiments revealed that during the fermentation of *B. subtilis* at 36°C (Figure 3A), the alkaline protease activity reached a stable phase with a remarkable enzyme activity of 758 U/mL. This activity was significantly higher than that observed at 28°C and 32°C. However, an increase beyond 36°C resulted in a gradual decline in the alkaline protease activity. Among other investigated parameters, the highest alkaline protease activity of 600 U/mL was achieved at an agitation speed of 180 rpm (Figure 3B). Furthermore, when the inoculation volume was set at 8% (Figure 3C), the fermentation broth exhibited an optimal alkaline protease activity of 580 U/mL. Additionally, an initial pH of 8.0 (Figure 3D) yielded the maximum alkaline protease activity, measuring 640 U/mL. Moreover, the alkaline protease activity reached its peak value of 670 U/mL when the airflow rate was maintained at 1:1.2 vvm (Figure 3E). Consequently, the findings suggest that a fermentation temperature of 36°C, an inoculation volume of 8%, an initial pH of 8.0, an agitation speed of 180 rpm and an airflow rate of 1:1.2 vvm are considered optimal conditions for alkaline protease accumulation by *B. subtilis*.

### 3.3 The rapid screening of significant factors influencing enzyme activity

Based on the single-variable experiments, a comprehensive investigation of the interaction effects of variables, including corn starch (A), corn syrup powder (B), MgSO_4_ (C), MnSO_4_ (D), agitation speed (E), fermentation temperature (F), airflow rate (G), inoculation volume (H), and initial pH (I), was performed according to the Plackett–Burman design in Table 1. As resulted in Supplementary Table S1, the enzyme activity responses for all experimental combinations exceeded 1,000 U/mL, reaching a maximum of 1,258 U/mL. In Table 2, the model (in terms of coded factors: *Y* = 1103.50 + 12.17A-38.17B-17.50C-16.50D-7.17E+15.83F-21.17G + 23.83H + 10.17I) established in this experiment exhibits extremely high significance with a *p*-value <0.01. Corn syrup powder, MgSO_4_, inoculation volume, and airflow rate had significant effects at a significance level of 0.01 < *p* < 0.05. However, the remaining factors do not have a significant effect on the production of alkaline protease by *B. subtilis* at a significance level of *p* < 0.05. Therefore, these four factors need to be further explored.

**Table 1:** The Plackett–Burman design.

Variables	Experiment levels	
	−1	+1
A: Corn starch (%)	0.3	0.7
B: Corn syrup powder (%)	0.5	1.5
C: MgSO_4_ (%)	0.04	0.06
D: MnSO_4_ (%)	0.04	0.06
E: Agitation speed (rpm)	170	190
F: Fermentation temperature (°C)	34	38
G: Airflow rate (vvm)	1:1	1:1.4
H: Inoculation volume (%)	6	10
I: Initial pH	7.0	9.0

**Table 2:** The analysis of variance (ANOVA) for the Plackett–Burman regression model.

Factors	Quadratic sum	Degree of freedom	Mean square	Regression coefficient	*F* value	*p*-value	Significance
Model	43256.33	9	4806.26	1103.50	24.99	0.0091	^**^
Corn starch	1776.33	1	1776.33	12.17	9.24	0.0934	
Corn syrup powder	17480.33	1	17480.33	−38.17	90.89	0.0108	^*^
MgSO_4_	3675.00	1	3675.00	−17.50	19.11	0.0486	^*^
MnSO_4_	3267.00	1	3267.00	−16.50	16.99	0.0541	
Agitation speed	616.33	1	616.33	−7.17	3.20	0.2153	
Fermentation temperature	3008.33	1	3008.33	15.83	15.64	0.0584	
Airflow rate	5376.33	1	5376.33	−21.17	27.95	0.0340	^*^
Inoculation volume	6816.33	1	6816.33	23.83	35.44	0.0271	^*^
pH	1240.33	1	1240.33	10.17	6.45	0.1263	

^*^Denotes notable differences (0.01 < *p* < 0.05). ^**^Denotes very notable differences (*p* < 0.01).

### 3.4 Response surface methodology and experimental verification

In Supplementary Table S2, these four significant factors, namely corn syrup powder (X_1_), MgSO_4_ (X_2_), inoculation volume (X_3_) and airflow rate (X_4_), were selected to conduct Box–Behnken design, aiming to explore the fermentation process of *B. subtilis* in the production of alkaline protease. Other variables were maintained at constant levels: 0.5% corn starch, 0.05% MnSO_4_, fermentation temperature of 36°C, agitation speed of 180 rpm, and initial pH of 8.0. The Supplementary Table S3 obtained the results that the enzyme activity response values of most experimental groups exceeded 1,000 U/mL. Overall, when all four variables are set to the 0 level, the enzyme activity response value is approximately 1700, indicating a relatively optimal combination. Furthermore, the variance analysis of the regression model demonstrated that the regression equation prediction model can be represented as:


$$\begin{array}{l} Y=1778.6+34.{\mathrm{5X}}_{1}-79.17X_{2}+19.92X_{3}+75.25X_{4}+93.25X_{1}X_{2}\\ \;\;\;\;\;\;\;\;\;-12{\mathrm{4X}}_{1}X_{3}-0.75X_{1}X_{4}+9.{\mathrm{5X}}_{2}X_{3}+61.25X_{2}X_{4}-48.25\;X_{3}X_{4}\\ \;\;\;\;\;\;\;\;\;-235.{\mathrm{8X}}_{1}{}^{2}-329.{\mathrm{8X}}_{2}{}^{2}-351.42X_{3}{}^{2}-326.68\;X_{4}{}^{2} \end{array}$$

The significance of the experimental model’s effect on the response value *Y* is evident from Table 3, as indicated by its extremely low *p*-value (<0.0001), thus providing high credibility. The lack-of-fit item in the model exhibits a *p*-value of 0.7504, which is greater than the significance level of 0.05, implying that the model fit is satisfactory. Based on the *p*-values, it can be concluded that all square terms have highly significant effects on the activity of alkaline protease produced by *B. subtilis* (*p* < 0.01). This result once again confirms the significant impact of these four factors, among the nine factors studied, on the accumulation of alkaline protease. The linear terms of corn syrup powder, MgSO_4_, inoculation volume, and airflow rate have corresponding *F* values of 1.35, 7.10, 0.45, and 6.93, respectively. These values suggest that the factors influencing the accumulation of alkaline protease follow the order: MgSO_4_ > airflow rate > corn syrup powder > inoculation volume. When considering the *F* value and *p*-value of both the linear and quadratic terms, the influence of MgSO_4_ and airflow rate on enzyme activity is also likely to be more prominent compared to the inoculation volume and corn syrup powder. Additionally, the coefficient of determination (adjusted *R*^2^) for the model is 0.9294, demonstrating a strong correlation between the actual and predicted values. The estimated value of the predicted *R*^2^ is 0.7087, with a difference of 0.15 from the adjusted *R*^2^, which is less than 0.2. This indicates that the regression model can adequately explain the manufacturing process. A coefficient of variation (c.v.) value of 8.14% being below the threshold of 10% indicates high reliability and precision in the experiment. Furthermore, the residual diagnostic analysis provides the normal probability plot of residuals, the plot of residuals against predicted values, and the plot of predicted values against actual values. Supplementary Figure S2 shows that the normal probability plot of residuals is approximately linear, indicating a nearly normal distribution of residuals. The plot of residuals against predicted values does not exhibit any clear patterns, suggesting the absence of systematic errors. Additionally, the plot of predicted values against actual values also shows an ideal linear distribution. Overall, these findings indicate the accuracy of the regression model.

**Table 3:** The analysis of variance (ANOVA) for the Box–Behnken regression model.

Sources	Quadratic sum	Degree of freedom	Mean square	*F* value	*p*-value	Significance
Models	1.952 × 10^6^	14	1.394 × 10^5^	13.16	<0.0001	^**^
X_1_	14283.00	1	14283.00	1.35	0.2651	
X_2_	75208.33	1	75208.33	7.10	0.0185	^*^
X_3_	4760.08	1	4760.08	0.45	0.5136	
X_4_	73476.75	1	73476.75	6.93	0.0197	^*^
X_1_X_2_	34782.25	1	34782.25	3.28	0.0915	
X_1_ X_3_	61504.00	1	61504.00	5.80	0.0303	^*^
X_1_X_4_	2.25	1	2.25	2.123 × 10^−4^	0.9886	
X_2_ X_3_	361.00	1	361.00	0.034	0.8562	
X_2_X_4_	15006.25	1	15006.25	1.42	0.2538	
X_3_X_4_	9312.25	1	9312.25	0.88	0.3644	
X_1_^2^	3.607 × 10^5^	1	3.607 × 10^5^	34.03	<0.0001	^**^
X_2_^2^	7.055 × 10^5^	1	7.055 × 10^5^	66.58	<0.0001	^**^
X_3_^2^	8.011 × 10^5^	1	8.011 × 10^5^	75.60	<0.0001	^**^
X_4_^2^	6.922 × 10^5^	1	6.922 × 10^5^	65.32	<0.0001	^**^
Residual	1.484 × 10^5^	14	10596.77			
Lack of fit	90533.58	10	9053.36	0.63	0.7504	
Pure error	57821.20	4	14455.30			
Cor. total	2.100 × 10^6^	28				
*R* ^2^	0.9294					
Adjusted *R*^2^	0.8587					
C.V. %	8.14					
Adeq precision	11.8207					

^*^Denotes notable differences (0.01 < *p* < 0.05). ^**^Denotes very notable differences (*p* < 0.01).

**Figure 4:**
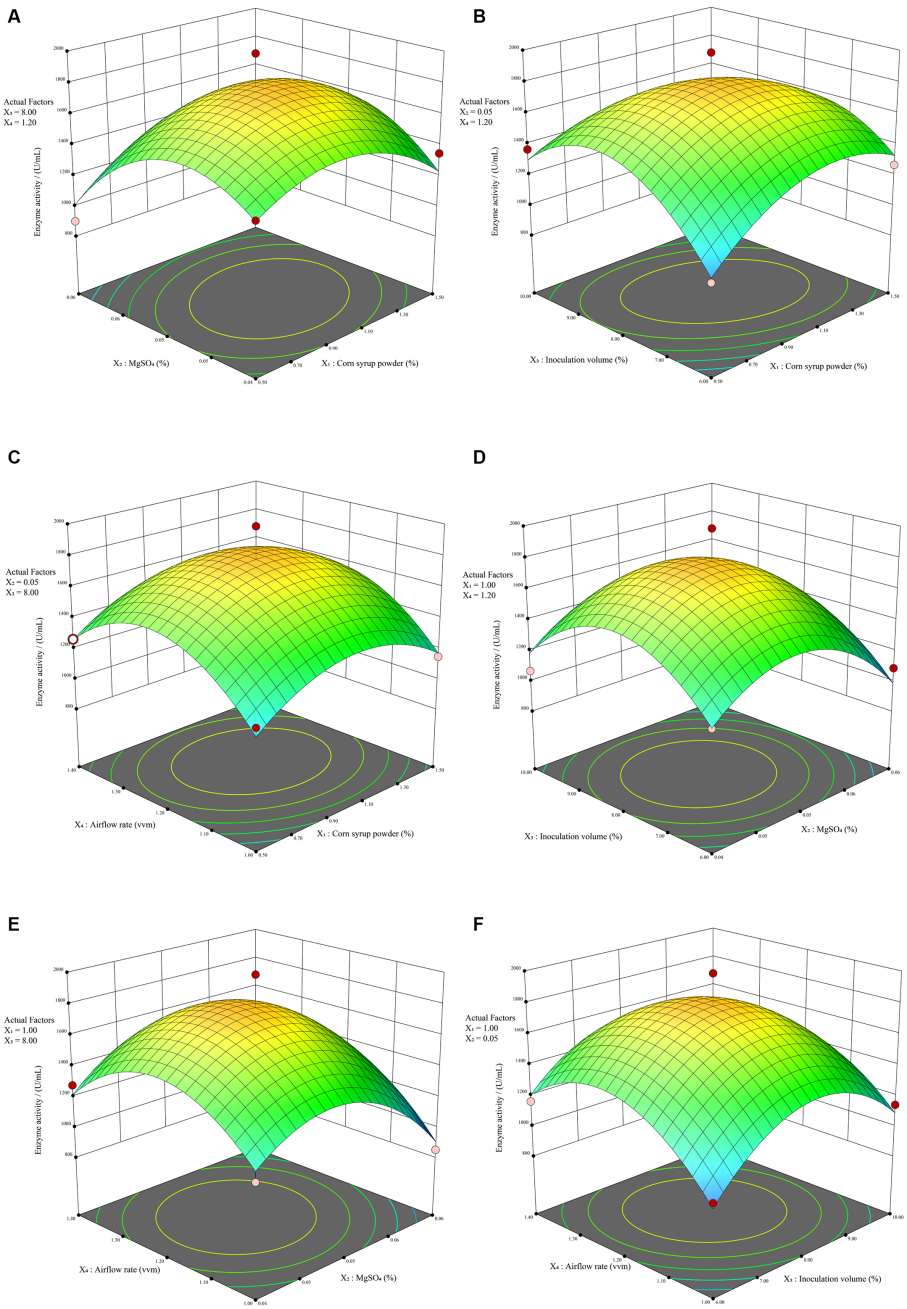
The RSM results. The response surface plot illustrating the impact of the interaction between corn syrup powder and MgSO_4_ **(A)**, corn syrup powder and inoculation volume **(B)**, corn syrup powder and airflow rate **(C)**, MgSO_4_ and inoculation volume **(D)**, MgSO_4_ and airflow rate **(E)**, inoculation volume and airflow rate **(F)** on enzyme activity.

RSM overcomes the limitation of orthogonal experiments, which do not provide intuitive visual representations. It involves creating three-dimensional response surface plots and contour plots based on quadratic equations to illustrate the interactions between experimental factors. Generally, response surface plots and contour plots visually represent the impact of interactions on response values. Steeper surfaces and denser contour lines indicate more significant effects, and when contour lines form ellipses, it signifies a stronger interaction between the two factors. As depicted in Figure 4B, it indicates a significant interaction (*p* < 0.05) between corn syrup powder (X_1_) and inoculation volume (X_3_), resulting in a steeper response surface. This observation is consistent with the contour plot presented in Supplementary Figure S3B, where a discernible elliptical pattern is observed, indicating the presence of an interaction effect between the two factors. The contour plots of corn syrup powder (X_1_) and MgSO4 (X_2_) exhibit an elliptical trend. Although the regression model shows a *p*-value of 0.0915 > 0.05, not reaching the significance threshold, there still exists a certain degree of interaction effect (Supplementary Figure S3A). Except for these two combinations, the remaining four combinations did not exhibit strong interaction effects. The contour plots of the four combinations (Supplementary Figures S3C–F) all show approximately circular patterns. In contrast, the three-dimensional response surface plots for all six combinations (Figure 4) display distinct steep trends, indicating a significant impact of the four factors on alkaline protease. Overall, the experimental model effectively captures the variations in enzyme activity of alkaline protease produced by *B. subtilis* under different conditions. Consequently, it can be employed for the analysis and prediction of alkaline protease enzyme activity in *B. subtilis* fermentation.

Finally, based on the optimal model mentioned above, the recommended combination of 1.03% corn syrup powder, 0.05% MgSO_4_, 8.02% inoculation volume, and 1:1.22 vvm airflow rate was adopted in the confirmatory trial. The other variables used for fermentation verification were maintained at 0.5% corn starch, 0.05% MnSO_4_, a fermentation temperature of 36°C, an agitation speed of 180 rpm and an initial pH of 8.0. As a result, the measured activity of alkaline protease during the verification was 1780.03 U/mL, which closely matched the predicted value of 1787.91 U/mL, demonstrating the reliability of the mathematical model established in this research.

### 3.5 The 500 L scale-up fermentation of *Bacillus subtilis* for producing alkaline protease

**Figure 5:**
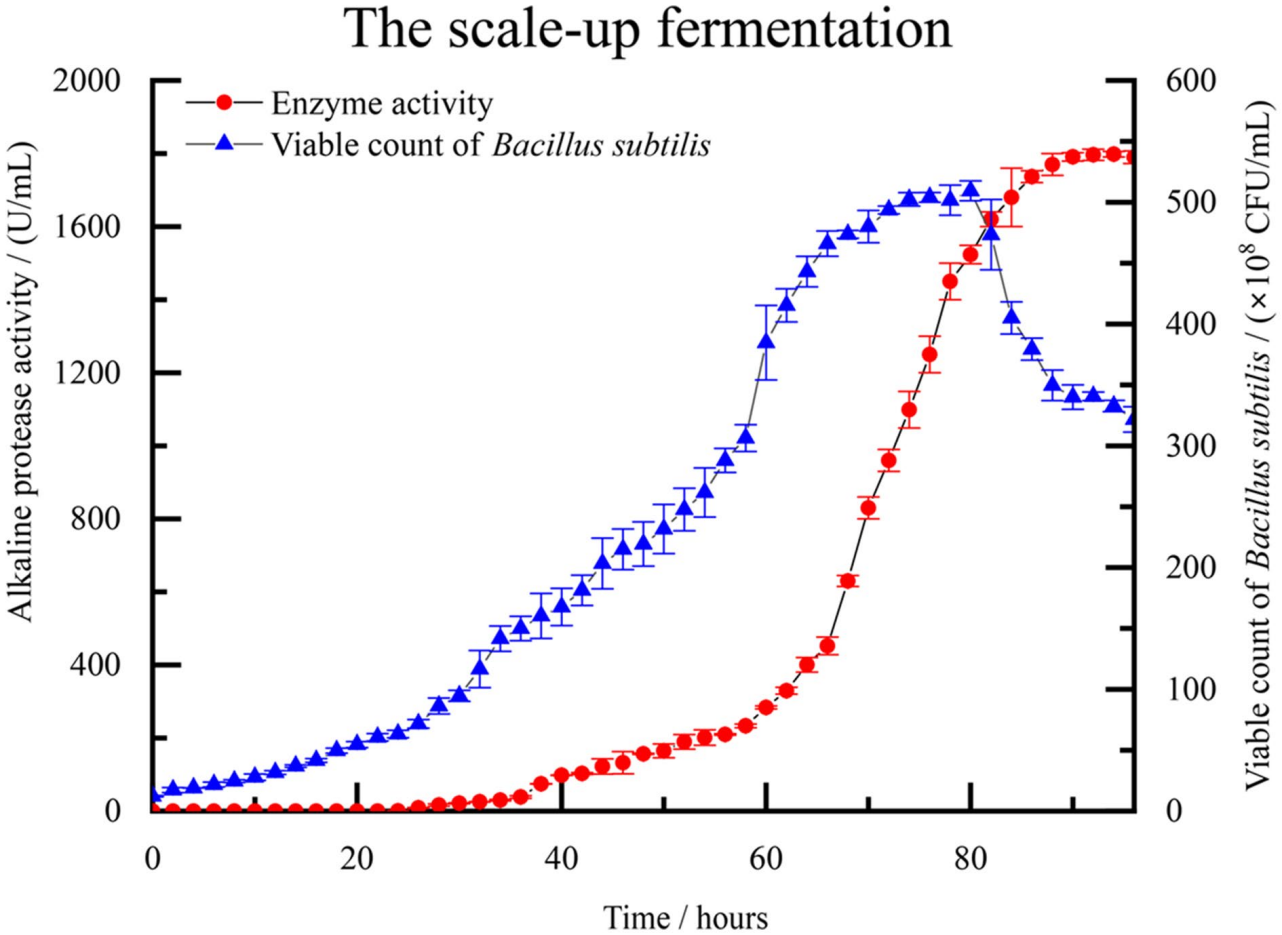
The fermentation curve of *B. subtilis* in 500 L scale-up fermentation. Enzyme activity is represented by solid red circles, while the count of viable *B. subtilis* spores is represented by solid blue triangles.

A scale-up test was conducted based on the previous fermentation optimization, using a 500 L fermentation system. Figure 5 illustrates the monitoring of control parameters throughout the fermentation test. The observed changes further validated the repeatability of the experimental results. During the initial 0–24 h, the population of *B. subtilis* increased slowly, and the enzyme activity remained close to 0, indicating a lag phase of the strain in the new medium environment. After 26 h, the number of viable bacteria in the fermentation broth began to increase rapidly. At about 70 h, the number of viable bacteria is close to the equilibrium period, about 490–500 × 10^8^ CFU/mL. Concurrently, the enzyme activity of alkaline protease started to rise rapidly, along with the accumulation of secondary metabolites. By the 94 h mark, the enzyme activity reached 1798.33 U/mL, which signifies that the enzymatic activity of the alkaline protease after optimization reached 300.72% of the pre-optimized level. As the culture time extended, the number of viable bacteria in the fermentation broth gradually declined, accompanied by a significant production of spores. This period corresponded to the depletion of nutrients in the fermented liquid.

## 4 Discussion

### 4.1 *Bacillus subtilis* BS-QR-052 begins to accumulate alkaline protease at the beginning of the logarithmic phase and continues until the stationary phase or decline phase

Numerous studies have indicated that in most cultivation conditions, species of *Bacillus*, like *Bacillus subtilis*, produce extracellular alkaline proteinases during the late exponential and stationary phases of batch culture growth (Singh et al., 2004; Zhou et al., 2019; Jafari et al., 2023). This pattern aligns with the consistency observed in our study, both before and after the optimization of the medium and fermentation conditions. Furthermore, other microbial species, such as *Pseudomonas aeruginosa* (Saleem, 2012), also exhibit similar fermentation patterns when synthesizing alkaline proteases. The model demonstrates that in *Bacillus* fermentation processes with shorter cycles (10–30 h), the peak activity of alkaline protease occurs 2–12 h after the point of maximum biomass (Singh et al., 2004; Subba Rao et al., 2009), while in longer fermentation cycles (60–80 h), the peak activity of alkaline protease lags behind the time of highest cell density by 30–38 h (Saleem, 2012; Jafari et al., 2023). However, the stage of the cell growth cycle at which extracellular protease synthesis reaches its maximum has been a topic of ongoing debate. The situations can be categorized into two classes (Priest, 1977): (i) during the active growth phase, the secretion rate of extracellular proteases is very low, and subsequently increases during the late exponential and early stationary phases of growth; (ii) the synthesis and secretion of extracellular enzymes increase with growth and decrease as the culture enters the stationary phase. The possible reasons for this may be attributed to different carbon sources (Herbert, 1961) and spores (Mao et al., 1992). Cells cultured on a single carbon source experience a rapid decrease in metabolic activity and RNA content as they transition from the exponential to the stationary phase. However, when complex organic compounds are used as carbon or nitrogen sources, cells in the late exponential and early stationary phases can maintain a certain level of metabolic activity and RNA content, leading to continued accumulation of proteases (Herbert, 1961). Hence, in this research, the use of complex organic carbon and nitrogen sources is the primary factor contributing to this phenomenon.

Although this study resulted in a reduction of approximately 30 h in the fermentation cycle before and after optimization, and the delay in alkaline protease accumulation compared to cell proliferation was reduced from 20 h to 10 h, the overall fermentation cycle remains long at approximately 90 h. Generally, the synthesis and accumulation of microbial extracellular proteases are strongly influenced by the composition of the culture medium (Bhunia et al., 2012). Factors such as the C/N (carbon-to-nitrogen) ratio, the presence of easily metabolizable sugars (such as glucose) and metal ions (e.g., Mg^2+^ and Ca^2+^), and the availability of rapidly utilizable nitrogen sources, like amino acids, have been confirmed to influence on the synthesis of alkaline protease significantly. In this research, corn starch was used as a carbon source to avoid glucose catabolic repression. Corn syrup powder is used as an organic nitrogen source, which can promote higher protease production and avoid the inhibitory effect of rapidly available nitrogen sources on protease synthesis. The use of organic carbon and nitrogen sources reduces costs and avoids metabolic inhibition, but it prolongs the production cycle to a certain extent, which may be the main reason why the fermentation cycle lasted more than 90 h in this study.

### 4.2 Nutrition and conditions are important factors affecting the production of alkaline protease by *Bacillus subtilis* BS-QR-052

High productivity is one of the advantages of *B. subtilis* in producing alkaline protease. The enzyme activity of alkaline protease produced by wild strains ranges from 50 to 500 U/mL (Nisha and Divakaran, 2014; Bukhari et al., 2020), and can be increased to 500–1,000 U/mL through optimization of the culture medium or overexpression methods (Shafique et al., 2021; Emon et al., 2023). In this study, the enzyme activity was 598 U/mL and 1780.03 U/mL before and after optimization, respectively, representing a remarkable 198% improvement. While the fold increase achieved may be slightly lower compared to other process optimization strategies (Ozdenefe et al., 2017), the enzyme activity still reached a significantly high level. Additionally, implementing measures such as fed-batch fermentation (Chen et al., 2022) can further enhance the production of alkaline protease, highlighting the productivity advantage of *B. subtilis* in the field of alkaline protease production. Therefore, selecting suitable nutrients and optimizing various process conditions are crucial approaches to maximize the yield of alkaline protease produced through *B. subtilis* fermentation.

### 4.3 Composite sources are more effective, and inorganic salts and metal ions are essential

The cultivation medium for alkaline protease production in *B. subtilis* typically comprises carbon sources, nitrogen sources, inorganic salts, and metal ions. Common carbon sources include glucose, sucrose, lactose, maltose, corn starch, maltodextrin, and cornmeal. Nitrogen sources commonly used are soybean meal, peptone, urea, yeast extract, beef paste, soybean cake powder, and corn syrup powder. Previous research suggests that a combination of carbon and nitrogen sources may be more suitable for enhancing the fermentation process and maximizing alkaline protease production by *B. subtilis* (Krishnaveni et al., 2012; Nisha and Divakaran, 2014; Sathishkumar et al., 2015). In this study, we employed a composite carbon source (corn starch) and a composite nitrogen source (corn syrup powder) to optimize the production yield of alkaline protease. Through single-variable optimization, we achieved an impressive nearly 1-fold increase in production yield. Notably, corn syrup powder, as a composite nitrogen source, emerged as a significant factor influencing the yield, closely linked to the secretion of extracellular protease. Additionally, the inclusion of inorganic salts and metal ions contributed to the maintenance of extracellular alkaline protease stability, aligning with the pronounced impact observed with the addition of MgSO_4_ (Nisha and Divakaran, 2014). Hence, the search for an optimal combination of cultivation medium components holds great promise in promoting the maximum secretion of extracellular alkaline protease by *B. subtilis*.

### 4.4 Statistical modeling and response surface methodology have significant potential applications in the optimization processes of biochemical systems

RSM stands out as a powerful tool in the optimization processes of chemical and biochemical systems. Its considerable success in the realm of biotechnological industrial production is evident in various reports. Notably, RSM has proven effective in optimizing the production of microbial-synthesized products, including enzymes, pigments, and pectin oligosaccharides (Breig and Luti, 2021). For example, *Lysinibacillus sphaericus* AA6 EMCCN3080 achieved a remarkable 98-fold increase in alkaline protease (ALK-PR23) activity through RSM optimization (Matrawy et al., 2023b). In our study, a similar approach significantly enhanced alkaline protease activity, reaching an impressive 1798.33 U/mL. Other instances include the 7.58-fold increase in α-amylase (CA-AM21) yield in *Glutamicibacter soli* AM6 EMCCN 3074 (Matrawy et al., 2023a), and the 8.95 and 5-fold production boosts in xylanase for *Penicillium chrysogenum* A3 DSM105774 (Matrawy et al., 2021b) and *Thermomyces lanuginosus* A3-1 DSM 105773 (Matrawy et al., 2021a), respectively. Furthermore, the pectinase from *Aspergillus* exhibited a 13-fold improvement in producing pectin oligosaccharides during citrus pectin fermentation (Embaby et al., 2016). In the biotransformation of natural pigments, the application of RSM significantly elevated the pigment production efficiency in *Monascus purpureus* ATCC 16436 strain (Embaby et al., 2018). These findings underscore the versatile and impactful role of RSM in optimizing diverse biochemical processes for enhanced biotechnological outcomes.

## 5 Conclusion

In this study, a series of experiments were conducted to optimize the fermentation medium composition and process conditions for the production of *B. subtilis* BS-QR-052 alkaline protease. The results indicate that corn starch and corn syrup powder are suitable carbon and nitrogen sources, while the presence of Mg^2+^ and Mn^2+^ positively influences the production of alkaline protease. Moreover, specific process conditions were identified as optimal: a fermentation temperature of 36°C, an inoculation volume of 8%, an initial pH of 8.0, an agitation speed of 180 rpm, and an airflow rate of 1:1.2 vvm. Notably, the Plackett-Burman design revealed that corn syrup powder, MgSO_4_, inoculation volume, and airflow rate had significant impacts on the accumulation of alkaline protease. Through RSM, a prediction model was simulated, and the optimal values were predicted. Subsequently, the model was experimentally validated, and after optimization, the enzyme’s activity increased to 300.72% of its original level. This indicates that the findings of this study possess certain stability and repeatability, laying the foundation for further production and promotion of alkaline protease.

## Data availability statement

The original contributions presented in the study are included in the article/Supplementary material, further inquiries can be directed to the corresponding author.

## Author contributions

BS: Conceptualization, Funding acquisition, Methodology, Project administration, Resources, Writing – original draft. KZ: Conceptualization, Data curation, Investigation, Methodology, Project administration, Validation, Visualization, Writing – original draft, Writing – review & editing. YiZ: Data curation, Investigation, Methodology, Project administration, Resources, Validation, Writing – review & editing. YT: Conceptualization, Investigation, Methodology, Writing – review & editing. FZ: Formal analysis, Funding acquisition, Project administration, Resources, Supervision, Writing – review & editing. WC: Formal analysis, Project administration, Supervision, Writing – review & editing. XT: Data curation, Methodology, Validation, Writing – review & editing. CC: Data curation, Methodology, Validation, Writing – review & editing. YaZ: Data curation, Methodology, Validation, Writing – review & editing.
