# Supplementary material for: The fermentation optimization for alkaline protease production by Bacillus subtilis BS-QR-052
Source: Front Microbiol. 2023 Dec 19;14:1301065. doi: 10.3389/fmicb.2023.1301065 (PMC10758460; doi:10.3389/fmicb.2023.1301065)
Supplement: Supplementary file 1 [file Table_1.DOCX]

**Supplementary Tables**

**Table S1. The response to Plackett-Burman design**

| **NO.** | **Corn starch** | **Corn syrup powder** | **MgSO_4_** | **MnSO_4_** | **Fermentation temperature** | **Agitation speed** | **Inoculation volume** | **pH** | **Airflow rate** | **Enzyme activity (U/mL)** |
| --- | --- | --- | --- | --- | --- | --- | --- | --- | --- | --- |
| 1 | -1 | -1 | +1 | -1 | +1 | +1 | -1 | +1 | +1 | 1102 |
| 2 | +1 | +1 | -1 | -1 | -1 | +1 | -1 | +1 | +1 | 1054 |
| 3 | +1 | -1 | -1 | -1 | +1 | -1 | +1 | +1 | -1 | 1258 |
| 4 | -1 | +1 | +1 | +1 | -1 | -1 | -1 | +1 | -1 | 1010 |
| 5 | -1 | +1 | +1 | -1 | +1 | +1 | +1 | -1 | -1 | 1096 |
| 6 | -1 | -1 | -1 | +1 | -1 | -1 | +1 | -1 | +1 | 1092 |
| 7 | -1 | -1 | -1 | -1 | -1 | -1 | -1 | -1 | -1 | 1150 |
| 8 | +1 | -1 | +1 | +1 | +1 | -1 | -1 | -1 | +1 | 1088 |
| 9 | +1 | +1 | +1 | -1 | -1 | -1 | +1 | -1 | +1 | 1060 |
| 10 | +1 | +1 | -1 | +1 | +1 | +1 | -1 | -1 | -1 | 1074 |
| 11 | -1 | +1 | -1 | +1 | +1 | -1 | +1 | +1 | +1 | 1098 |
| 12 | +1 | -1 | +1 | +1 | -1 | +1 | +1 | +1 | -1 | 1160 |

**Table S2. The Box-Behnken Design**

| Variables | Experiment levels | | |
| --- | --- | --- | --- |
|  | **-1** | **0** | **+1** |
| Corn syrup powder (X_1_) | 0.5% | 1% | 1.5% |
| MgSO_4_ (X_2_) | 0.04% | 0.05% | 0.06% |
| Inoculation volume (X_3_) | 6% | 8% | 10% |
| Airflow rate (X_4_) | 1:1.0 vvm | 1:1.2 vvm | 1:1.4 vvm |

**Table S3. The response of Box-Behnken design**

| **No.** | **Corn syrup powder** | **MgSO_4_** | **Inoculation volume** | **Airflow rate** | **Enzyme activity (U/mL)** |
| --- | --- | --- | --- | --- | --- |
| 1 | -1 | 0 | +1 | 0 | 1370 |
| 2 | -1 | 0 | -1 | 0 | 985 |
| 3 | +1 | 0 | +1 | 0 | 1158 |
| 4 | 0 | +1 | 0 | +1 | 1255 |
| 5 | 0 | 0 | 0 | 0 | 1756 |
| 6 | 0 | -1 | 0 | -1 | 1120 |
| 7 | -1 | 0 | 0 | -1 | 1156 |
| 8 | +1 | 0 | 0 | +1 | 1256 |
| 9 | +1 | 0 | -1 | 0 | 1269 |
| 10 | +1 | -1 | 0 | 0 | 1350 |
| 11 | 0 | -1 | 0 | +1 | 1280 |
| 12 | -1 | +1 | 0 | 0 | 900 |
| 13 | -1 | -1 | 0 | 0 | 1358 |
| 14 | 0 | +1 | +1 | 0 | 1032 |
| 15 | 0 | 0 | -1 | -1 | 989 |
| 16 | 0 | +1 | -1 | 0 | 1088 |
| 17 | -1 | 0 | 0 | +1 | 1265 |
| 18 | 0 | 0 | +1 | +1 | 1126 |
| 19 | 0 | 0 | 0 | 0 | 1689 |
| 20 | +1 | +1 | 0 | 0 | 1265 |
| 21 | 0 | -1 | +1 | 0 | 1069 |
| 22 | 0 | +1 | 0 | -1 | 850 |
| 23 | 0 | 0 | 0 | 0 | 1985 |
| 24 | 0 | 0 | 0 | 0 | 1765 |
| 25 | 0 | 0 | +1 | -1 | 1143 |
| 26 | +1 | 0 | 0 | -1 | 1150 |
| 27 | 0 | -1 | -1 | 0 | 1163 |
| 28 | 0 | 0 | 0 | 0 | 1698 |
| 29 | 0 | 0 | -1 | +1 | 1165 |
