# Supplementary material for: The fermentation optimization for alkaline protease production by Bacillus subtilis BS-QR-052
Source: Front Microbiol. 2023 Dec 19;14:1301065. doi: 10.3389/fmicb.2023.1301065 (PMC10758460; doi:10.3389/fmicb.2023.1301065)
Supplement: Supplementary file 2 [file Data_Sheet_1.DOCX]

**Supplementary Figures**

**
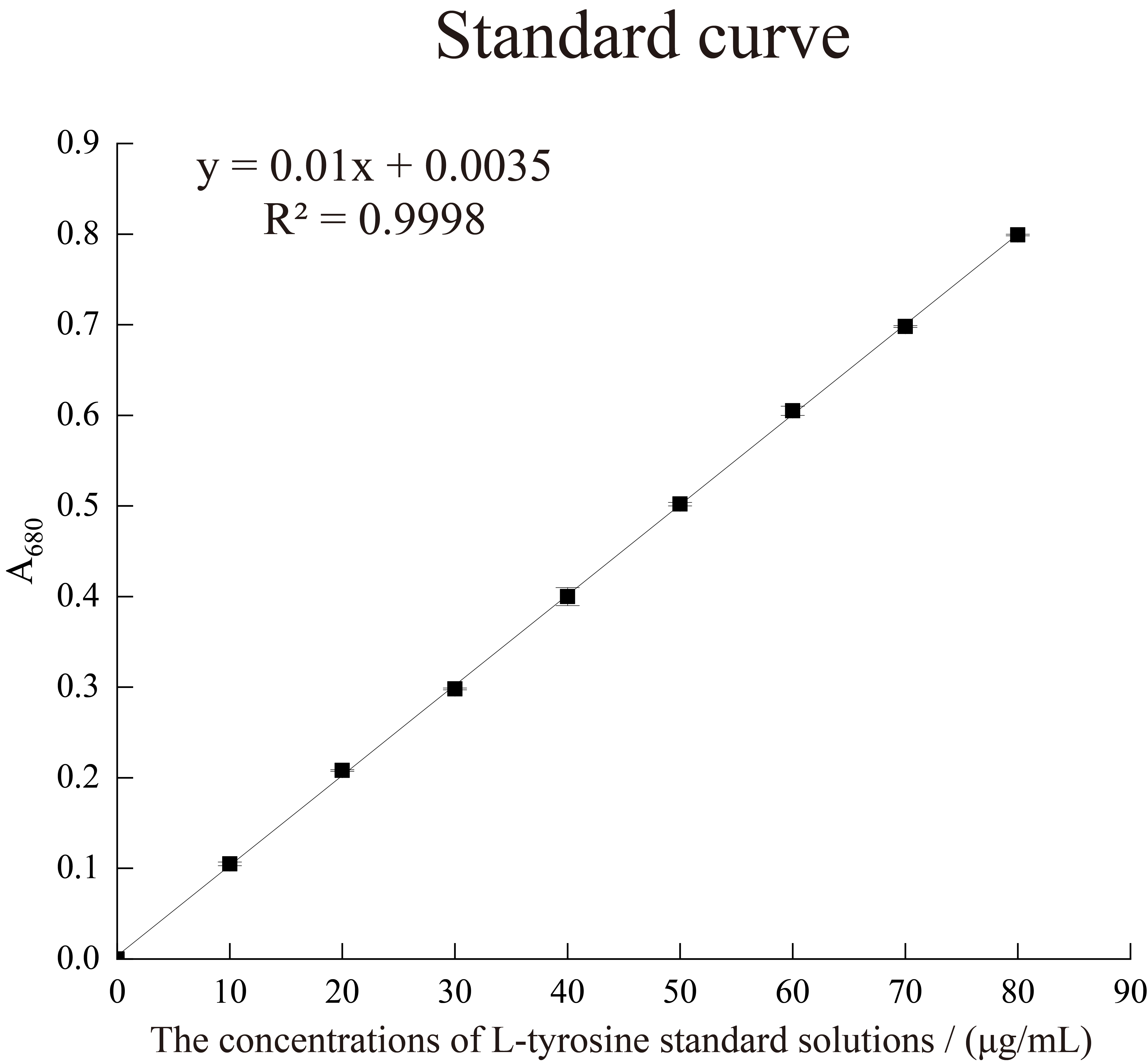
**

**Figure S1. The standard curve for determining the alkaline protease activity.**

**

**

**Figure S2. The diagnostic analysis of residuals.** (A) The normal plot of residuals. (B) The residuals vs. predicted. (C) The predicted vs. actual.

**

**

**Figure S3. The contour plot of the response surface analysis.** The contour plot illustrating the impact of the interaction between corn syrup powder and MgSO_4_ (**A**), corn syrup powder and inoculation volume (**B**), corn syrup powder and airflow rate (**C**), MgSO_4_ and inoculation volume (**D**), MgSO_4_ and airflow rate (**E**), inoculation volume and airflow rate (**F**) on enzyme activity.
